# Synthesis and Properties of Gelatin Methacryloyl (GelMA) Hydrogels and Their Recent Applications in Load-Bearing Tissue

**DOI:** 10.3390/polym10111290

**Published:** 2018-11-21

**Authors:** Mingyue Sun, Xiaoting Sun, Ziyuan Wang, Shuyu Guo, Guangjiao Yu, Huazhe Yang

**Affiliations:** 1 School of Fundamental Sciences, China Medical University, Shenyang 110122, China; smilesmy@foxmail.com (M.S.); xtsun@cmu.edu.cn (X.S.); 2 The Queen’s University of Belfast Joint College, China Medical University, Shenyang 110122, China; zwang19@qub.ac.uk (Z.W.); Sguo04@qub.ac.uk (S.G.); gyu01@qub.ac.uk (G.Y.)

**Keywords:** GelMA, photocrosslink, cell culture, load-bearing tissue

## Abstract

Photocrosslinked gelatin methacryloyl (GelMA) hydrogels have attracted great concern in the biomedical field because of their good biocompatibility and tunable physicochemical properties. Herein, different approaches to synthesize GelMA were introduced, especially, the typical method using UV light to crosslink the gelatin-methacrylic anhydride (MA) precursor was introduced in detail. In addition, the traditional and cutting-edge technologies to characterize the properties of GelMA hydrogels and GelMA prepolymer were also overviewed and compared. Furthermore, the applications of GelMA hydrogels in cell culture and tissue engineering especially in the load-bearing tissue (bone and cartilage) were summarized, followed by concluding remarks.

## 1. Introduction

Hydrogels possess three-dimensional hydrophilic polymer networks, which can swell in water and become much larger than their initial weight without dissolution. In order to be applied in various fields, tunable physical and chemical properties are necessary for hydrogels. Their physicochemical properties can be altered by combinations of comonomers, the crosslink density, and varying synthetic conditions (such as reaction time, temperature, and types and dosage of solvent and so on.) [1]. Hydrogels play an important role in bio-medical fields [1,2,3,4,5], such as tissue engineering, regenerative medicine, drug delivery and so forth. In addition, hydrogels are highly similar to the natural extracellular matrix [4], which can offer mimetic conditions for in vitro cell culture. Hydrogels are mainly divided into natural hydrogels and synthetic hydrogels [2]. Compared with synthetic hydrogels, natural hydrogels have better biocompatibility, which significantly contributes to cellular viability, multiplication, differentiation, and locomotion [5]. With the development of material science, hydrogel-based scaffolds for cell culture have great potential in the field of tissue engineering.

Gelatin is a kind of natural hydrophilic polymer produced from hydrolysis and denaturation of collagen under high temperature [6]. Gelatin possesses a series of advantages, including good biocompatibility, solubility, degradability, easy acquirement and so on. [6]. In particular, compared with collagen, the antigenicity of gelatin is lower. Moreover, gelatin keeps up the arginine-glycine-aspartic acid (RGD) peptide sequence which favors certain cell behaviors (such as adhesion, proliferation, and differentiation) and a matrix metalloproteinase (MMP) degradation sequence which promotes cell enzymatic degradation [7,8]. Nevertheless, the thermostability of gelatin is poor (gelatin will become solution due to cleavage of hydrogen bonds when the temperature is above 37 °C), and chemical crosslinkings may affect the biocompatibility of gelatin because some crosslinking reagents are poisonous. Therefore, application of gelation is limited by the insufficient thermostability and potential poisonousness of chemical crosslinking. Fortunately, the side chains of gelatin have massive active groups, including –OH, –COOH, –NH_2_, –SH and so on. Therefore, it is possible for gelatin to be modified with specific groups to overcome its shortcomings.

GelMA was first mentioned in 2000 by Van Den Bulcke et al. [7]. GelMA is produced through the reaction of gelatin with methacrylic anhydride (MA) [7]. A large number of amino groups presenting on the side chains of gelatin are replaced by methacryloyl groups in methacrylic anhydride, forming modified gelatin. However, this modified gelatin is similar to gelatin that its solid-liquid transition is also influenced by temperature. Modified gelatin obtains the feature of photocrosslinking because of the presence of methacryloyl groups. Water solution of modified gelatin crosslinks immediately under UV light when photoinitiator is added, giving rise to GelMA hydrogels which have excellent thermostability. GelMA hydrogel is still capable of supporting cell behaviors as the RGD and MMP sequences are maintained, also, the biocompatibility and degradation property of gelatin have not been influenced. Furthermore, physical and chemical properties of GelMA hydrogels can be tuned flexibly to meet the requirements of various applications.

Compared to the previous review [9], in this review, we will summarize preeminent works on GelMA hydrogels from different aspects, including preparation methods, characterization methods, and their applications in cell culture and tissue engineering. In particular, we will focus on the applications in load-bearing tissue engineering.

## 2. Synthesis and Characterization of GelMA Hydrogels

### 2.1. Synthesis

The traditional preparation method of GelMA was put forward by Van Den Bulcke et al. [7] in 2000. In general, this method involved a direct reaction between gelatin and methacrylic anhydride (MA). The resultant groups of this reaction comprise methacrylamide groups and methacrylate groups. Compared with methacrylate groups, the ratio of methacrylamide groups is increased. As a result, the full name of GelMA is gelatin methacryloyl, which is widely used among researchers. The whole preparation process was described as, firstly, gelatin was dissolved in phosphate buffered saline (PBS, pH = 7.4). After being completely dissolved, MA was added to the mixture, and stirred. Secondly, the mixed solution was diluted with PBS to stop the reaction, then, the diluted solution was put in dialysis membrane. The aim of the dialysis process was to remove poisonous and unreacted MA as well as other byproducts, after complete dialysis, ultrapure water was added in dialyzed solution. Finally, the ultima solution was freeze-dried for storage. GelMA hydrogels can be gained by exposing aqueous solution of GelMA prepolymer added with photoinitiator (Irgacure 2959) under ultraviolet (UV) light. The degrees of substitution of GelMA can be changed by varying the addition volume of MA. The degrees of substitution may affect the porosity, pore size, compressive modulus, and swelling behavior of the hydrogel. Therefore, other synthesis methods with enhanced degree of substitution were proposed. For example, Martineau L et al. [10] reported that the water-soluble dimethyl sulfoxide (DMSO) could be used as the solvent. This method significantly increased the degree of substitution using DMSO to reduce the contact between MA and water, and to cause hydrolysis; Lee BH et al. [11] put forward the replacement of PBS with carbonate-bicarbonate (CB) buffer to obtain GelMA with high degree of substitution. 

In addition, both UV and visible light can initiate photocrosslinking, and the common photoinitiators work within the UV range, however, it is well known that UV light would do harm to organisms. Although cell activity would not be affected in short term exposure, the usage of UV light may have potential long-term side effects on cells [12]. Therefore, the study of visible light induced crosslinking has attracted much concern. Wang Z et al. [13] presented a bioprinting system using visible light crosslinkable bioinks. They illustrated that the visible light crosslinking could be achieved by adding eosin Y based photoinitiator to the mixture of polyethylene glycol diacrylate (PEGDA) and GelMA hydrogels, but PEG-GelMA hydrogels could not enable the attaching and spreading of cells. Later, Wang Z et al. [14,15] reported that the EY-GelMA bioink could be used in cell attachment and diffusion. Erkoc P et al. [16] also developed a novel visible-light-induced photopolymerization procedure without additional crosslinkers for GelMA, where eosin Y was selected as a photoinitiator, triethanolamine (TEA) as an electron donor, and N-vinyl-2-pyrrolidinone (NVP) as an accelerator. Eosin Y, which has lower toxicity than Irgacure 2959 was chosen as the common visible light photoinitiator [17]. Also, visible light crosslinking eliminates the potential side effects of UV crosslinking. The UV light or visible light irradiation parameters can be easily adjusted to accommodate the system and investigate mechanism [18,19]. However, UV light or visible light has a common defect in that they both have a poor penetration depth into human tissue [20]. 

### 2.2. Characterization and Property Control

#### 2.2.1. Degree of Substitution

The degree of substitution of methacrylamide modified gelatin is proportional to the amount of added MA. ^1^H nuclear magnetic resonance spectrometer (^1^H NMR) is frequently used to confirm the replacement of free amino groups on gelatin by methacrylate groups, also, the degree of substitution of methacrylamide modified gelatin can be analyzed. It is necessary to respectively dissolve methacrylamide modified gelatin and gelatin in deuterium oxide (D_2_O, 10% *w*/*v*, for instance) at room temperature. The degree of substitution of methacrylamide modified gelatin can be evaluated by calculating the peak area ratio of modified amino groups to primary amino groups [21,22]. In general, the calculation formula of the degree of substitution is defined as:

DS = *S*’/*S*, (1)

where *S*’ represents the average integral area of modified amino groups (C=C bond); *S* represents the average integral area of primary amino groups (–CH–NH bond) [19].

Lee BH et al. [23] reported that TNBS (2,4,6-Trinitro benzene sulfonic acid) method (also known as Habeeb method) and ^1^H-NMR can be used to measure the degree of substitution. The measuring process included dissolving methacrylamide modified gelatin and gelatin in sodium bicarbonate buffer and TNBS solution was added, and then, hydrochloric acid and sodium dodecyl sulphate were used to stop the reaction. The reaction of TNBS and amino groups could generate trinitrobenzene (TNP) derivative which had a characteristic peak at 335 nm, so that the number of amino groups can be calculated [24]. The degree of substitution could be estimated according to the number of amino groups before and after the substitution of gelatin.

#### 2.2.2. Chemical Groups

Fourier-transform infrared spectroscopy (FTIR) can be used to exhibit the absorption peaks and frequencies of molecules or groups [10]. Specific chemical groups and the methacrylation of gelatin can be detected by FTIR spectrum [25]. In another word, FTIR spectrum was used to confirm whether GelMA prepolymer and GelMA hydrogels were synthesized successfully. In a typical measurement, the gelatin, GelMA prepolymer, GelMA hydrogel and KBr were mixed in a ratio of 1:100, and FTIR analysis was implemented after tabletting. Compared to pure gelatin, the amide bands of methacrylated gelatin shifted to higher frequencies, also, ester and vinyl bands might appear in the infrared spectra [10].

#### 2.2.3. Morphology

The structure and morphology of methacrylamide modified gelatin or GelMA are often characterized by scanning electron microscopy (SEM). GelMA hydrogels which have specific shape and volume need to be soaked in PBS to remove the unpolymerized components, and freeze dried. They were attached to the sample stage with conductive tape and vacuum spray of gold film was performed, before observation with a scanning electron microscopy (SEM).

Polyporous network structure is mostly observed with GelMA hydrogel under SEM. The porosity can be calculated by NIH Image J software based on SEM images. High porosity (high porosity support diffusion of oxygen and nutrients toward the cells and drainage of waste products from the matrix [26]) and suitable pore size (cell ingrowth required suitable pore size [26]) are favorable for cell culture, also the porosity and pore size can be adjusted. For instance, Van Vlierberghe S et al. [26,27] reported that experimental parameters during cryogenic treatment, including cooling speed, gradient of temperature, and final freezing temperature, had great effects on polyporous network structure of GelMA hydrogel; The degree of substitution also has influence on the pore size of GelMA hydrogel. Chen Y-C et al. [28] reported that the degree of substitution of amino group was inversely proportional to pore size; Celikkin N et al. [29] illustrated that the increase of the GelMA concentration could lead to the decrease of porosity and the average pore size of GelMA hydrogel.

#### 2.2.4. Stability

The stability of colloid dispersion could be reflected by the absolute value of zeta potential. Absolute value of zeta potential is proportional to the repulsive force between colloidal particles. Greater repulsive force between particles will lead to the reduced aggregation of particles, that is to say, the colloid dispersion is more stable. The surface electrical properties of the particles can be characterized by Zeta potential, which provides a basis for their modification. The zeta potential might be influenced by temperature, counterion, pH and so on [30]. The zeta potential of methacrylamide modified gelatin dissolved in deionized water could be measured using the zeta potential analyzer. Chen, YM et al. [31] carried out a control over cell behavior through manipulation of zeta potential. The area of cell diffusion, migration speed, migration distance and so on would change according to the relative value of zeta potential to the critical zeta potential.

#### 2.2.5. Wettability

The wettability of hydrogel can be characterized by measuring the contact angle between the surface of a hydrogel membrane and a liquid drop, which is a reflection of hydrophilicity or hydrophobicity of materials [32]. Usually, 5 μL of testing liquid was dropped onto the surface of the solid sample, and then the variation of contact angle between the drop and GelMA hydrogel with time was monitored [33]. It was reported that the increased surface wettability could increase the adhesion of human osteoblast, indicating that hydrophilic materials were more suitable for bone tissue engineering applications [34,35].

#### 2.2.6. Mechanical Properties

The mechanical properties of GelMA hydrogel can be measured by compression test and tensile test. The solution of methacrylamide modified gelatin is packed into a specific mold (such as a 8 mm diameter cylinder), and placed under UV light (360–480 nm, 6.9 mW/cm^2^) for 60 seconds, then, the shaped GelMA hydrogel would be incubated in DPBS for 24 h and kept at 37 °C with a constant temperature water bath [36]. A mechanical tester (Instron 5542, Instron, Boston, MA, USA) was used to compress or stretch the samples to obtain the stress-strain curve of GelMA hydrogel at the rate of 20% strain/min. Then, the corresponding compressive modulus and elastic modulus (using parallel samples and multiple measurements to calculate the average values) were calculated according to the selected strain interval [37].

The mechanical properties of GelMA hydrogel can be regulated by changing GelMA concentration and UV exposure time. Schuurman W et al. [38] demonstrated that the compression modulus of GelMA hydrogel became larger with the increase of the GelMA concentration or UV exposure time, however, the compression modulus and concentration were in power-law relations, and had an exponential relationship with time. The compression modulus can be controlled by regulating the methacrylation degree of GelMA as well. Chen Y-C et al. [28] found that the increase of the degree of methacrylation could enhance the compression modulus of GelMA hydrogel. Nichol JW et al. [36] also reported that mechanical properties were related to the degree of methacrylation and the GelMA concentration. Increasing the degree of methacrylation and maintaining the GelMA concentration would increase the compressive modulus of GelMA hydrogel and vice versa.

#### 2.2.7. Swelling Property

The swelling property of a hydrogel is evaluated by weighing method. GelMA hydrogels were prepared in the same way described above for mechanical property measurements. The prepared samples are freeze-dried, and then the dry weight of the samples is recorded by an electronic balance. Kamel Rahali demonstrated that the samples were soaked in a solvent (such as ultrapure water or pH 7.4 PBS) and weighed at intervals [25]. In this study, the swelling balance can be analyzed and the swelling ratio can be calculated. The swelling ratio can be calculated by the following formula [37]:

(2) $$\mathrm{swelling}\;\mathrm{ratio}=\frac{wet\;weight-dry\;weight}{dry\;weight}\times 100\%,$$

The swelling properties of hydrogel have a significant impact on the shape of GelMA hydrogel. Consequently, a number of literatures have focused on the swelling properties. Nichol JW et al. [36] studied the influence of GelMA concentration and the degree of substitution of GelMA hydrogel on the swelling ratios. As a result, the swelling ratios could be decreased by increasing the degree of substitution of GelMA hydrogel. Also, the swelling ratio declined with increasing GelMA concentration, which was likely due to the rise in crosslink densities as GelMA concentration increased, resulting in a decreased swelling ratio [37].

#### 2.2.8. Degradability

Hydrogel may exhibit an accelerated degradation behavior in aqueous environment. The degradation degree can be estimated by measuring the weight difference before and after incubation in a certain medium. For instance, Zhao X et al. [37] reported that the freeze-dried GelMA hydrogel could be dispersed in a 37 °C solution, which was made up of 500 μL of DPBS and 2 U/mL of collagenase type II. In this study, the GelMA hydrogel was taken out at the specified time, a filter paper was used to absorb the excess liquid on the surface. It was necessary to freeze-dry the hydrogels before weighing. The percentage of degradation of a specified time can be calculated according to the following formula:

(3) $$D=\frac{W_{0}-W_{t}}{W_{0}}\times 100\%,$$

where *D* is the percentage of degradation of the predetermined time points, *W*_0_ is the initial weight, and *W_t_* is the weight at the predetermined time points. Pepelanova I et al. [39] pointed that the degree of degradation should be measured according to the original wet weight and the weight of GelMA hydrogel at each time point in the enzyme solution. They found that the speed of degradation was inversely correlated with the degree of functionalization, and the kinetics of degradation could be controlled by the hydrogel concentration and the amount of enzyme.

#### 2.2.9. Viscoelasticity

The viscoelasticity of GelMA hydrogel can be characterized by dynamic shear oscillation measurement at small strain [7]. Rheology measurements based on oscillatory shear deformation modes are performed with a rheometer equipped with parallel rough plates. The rheometer can provide the elastic modulus *G’* (also called real modulus or storage modulus) and the viscous modulus *G”* (also called imaginary modulus or loss modulus), which can be used to evaluate the stability of GelMA hydrogel [25]. The measurement parameters of the rheometer may be different in the literature [7,39].

## 3. Applications of GelMA Hydrogels

### 3.1. Cell Culture

Photocrosslinked GelMA hydrogel has proper degradability, excellent biocompatibility, inherent biological activity, controllable physical and chemical properties. These features make GelMA hydrogel an ideal platform to study cellular responses, which can provide attachment sites and signaling cues to guide cell culture and growth. Therefore, GelMA hydrogel is an attractive candidate for mimicking native extracellular matrix (ECM) in cell culture. Compared with traditional 2D cell culture, three-dimensional (3D) cell culture based on microenvironment are closer to in vivo tissue. Hence, GelMA hydrogels have been widely used for 3D cell culture. Pepelanova I et al. [39] demonstrated that GelMA hydrogels could be engineered to act as 3D cell culture platforms. They found that a low polymer concentration of GelMA and a low UV dosage were necessary to provide a cell promoting microenvironment for mesenchymal stem cells (MSCs). MSCs exhibited higher viability in the hydrogels with a low polymer concentration of GelMA and a low UV dosage.

**Figure 1:**
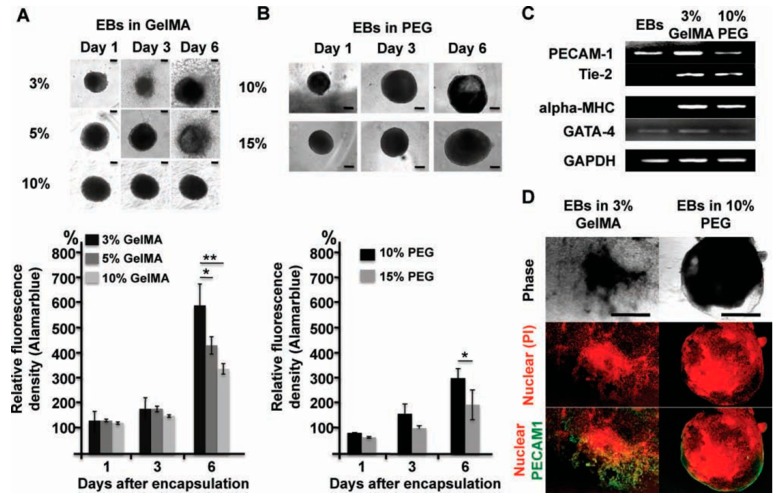
The effect of hydrogel properties on differentiation of EBs. (**A**,**B**) Phase contrast images of EBs. And cell proliferation was measured by using Alamarblue assay at same time point. (**C**) Expression of vasculogenic (PECAM1, Tie2) and cardiogenic (alpha-MHC and Gata4) in EBs after 6 days, and in EBs before encapsulation (left line). (**D**) Immunostaining PECAM1 (green) (scanning confocal microscopy) in EBs encapsulated in 3 wt % GelMA (**left** lane) and 10 wt % PEG (**right** lane) after 7 days culture with nuclear co-staining (PI, red) [40]. (Reproduced with permission from Qi H et al. Adv Mater; published by John Wiley and Sons, 2010).

Cell encapsulation can be achieved using photocrosslinked GelMA hydrogels. Microfabrication technique can also be used to encapsulate cells in GelMA hydrogel to control cell behaviors and functions. Qi H et al. [40] encapsulated embryoid bodies (EBs) into GelMA/polyethylene-glycol(PEG) hybrid microgels using micromolding and photolithography methods. They observed that proliferation and sprouting of EBs relied on the concentration of GelMA (Figure 1A), and spatially patterned vasculogenesis were achieved by adding the soluble chemical factors (Figure 1C,D). Ramón-Azcón J et al. [41] combined GelMA hydrogel with dielectrophoresis (DEP) method to develop a cell culture system. Grogan SP et al. [42] used GelMA hydrogel scaffolds to simulate the alignment of human meniscus cells in native meniscus tissue via projection printing method. GelMA hydrogel scaffolds, seeded with human meniscus cells for two weeks, were placed into the sites of meniscus defects in vitro. The results showed that GelMA hydrogel scaffolds could guide the alignment of human meniscus cells and the development of meniscus tissue in vitro. Jung J and Oh J also confirmed the possibility of bioencapsulation with GelMA hydrogel [43]. In this study, NIH/3T3 cells were encapsulated in the spherical GelMA microgels by microfluidic technique and photocrosslinking. By investigating the mechanical and swelling properties of the spherical GelMA microgels, as well as the viability of NIH/3T3 cells in the spherical GelMA microgels, the possibility of bioencapsulation had been demonstrated.

### 3.2. Applications in Load-Bearing Tissue

3D scaffold of GelMA hydrogels can guide the formation of a desired tissue due to their attachment sites and signaling cues. A variety of applications of GelMA hydrogels had been reported in tissue engineering, such as bones [44,45,46,47,48,49,50,51,52,53,54,55,56,57], endochondral bone [58,59], skin [60,61,62,63,64,65], myocardium [66], cardiac tissues [67,68,69,70,71,72], cartilage [42,73,74,75,76,77,78,79,80,81], vascular networks [82,83,84,85,86,87,88,89], skeletal muscle [90,91,92,93], cornea [94,95], interface [96] and so on. Ovsianikov A et al. [44] prepared 3D CAD scaffolds for tissue engineering applications using two-photon polymerization (2PP). They demonstrated that methacrylamide modified gelatin scaffolds prepared by 2PP method supported porcine MSC adhesion and differentiation into osteogenic lineage. 3D tissue engineering scaffolds could be manufactured by combining photosensitive methacrylamide modified gelatin with 2PP. Shin SR et al. [45] also reported a 3D scaffolding material for tissue engineering constructs using carbon nanotubes (CNT)–GelMA hybrid hydrogel system with tunable mechanical properties and improved cellular behavior. In this section, we mainly focused on the applications of GelMA hydrogels in load-bearing tissue, and the most innovative researches were summarized in Table 1. Also, studies which are closely related to or may provide valuable references to load-bearing tissue engineering were included [28,58,59,82,83,84,85,86,87,88,89,90,91,92,93]. For instance, Visser J et al. [58] found partial suppression of mineralization during the formation of endochondral bone, which is encouraging in the field of cartilage tissue engineering. Skeletal muscle is a muscle attached to the bone that controls the movement of bones. Enhancement of skeletal muscle formation is beneficial to regenerative medicine [93].

**Figure 2:**
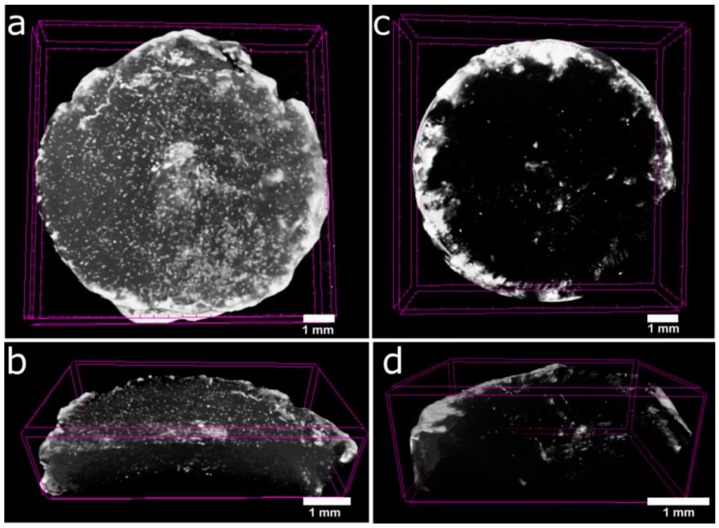
3D constructed µCT tomograms of GelMA_5% (**a**,**b**) and GelMA_10% (**c**,**d**) scaffolds from day 28 of in vitro culture with MSCs [29]. (Reproduced with permission from Celikkin N et al. Journal of Biomedical Materials Research; published by John Wiley and Sons, 2017).

Recently, the emergence of bone tissue engineering has been an area of concern for the repair of bone defects. The scaffold materials play an important role in the construction of tissue engineered bones. GelMA has already been used as scaffolds for bone regeneration in some bone tissue engineering studies. Celikkin. N et al. [29] reported that MSCs showed good osteogenic differentiation on GelMA hydrogels. They studied the effect of the GelMA concentration on MSCs osteogenic differentiation by evaluating the extracellular matrix calcification (Figure 2). The results showed that 5% GelMA hydrogel showed better bone tissue engineering performance over 10% GelMA hydrogel in vitro. In order to promote bone regeneration and repair, the osteogenic agents can be mixed into GelMA hydrogels. Heo DN et al. [97] embedded gold nanoparticles (GNPs) into the GelMA hydrogel by UV-induced chemical crosslinking. Thermo–chemical properties and enzymatic degradability of Gel–GNP were demonstrated, and they found that the alkaline phosphate (ALP) activity, proliferation, viability, and osteogenic differentiation of adipose-derived stem cells (ADSCs) were promoted in the Gel–GNP scaffold. The Gel–GNP scaffold can be used as an implant material for repairing bone defects according to the experimental results.

**Figure 3:**
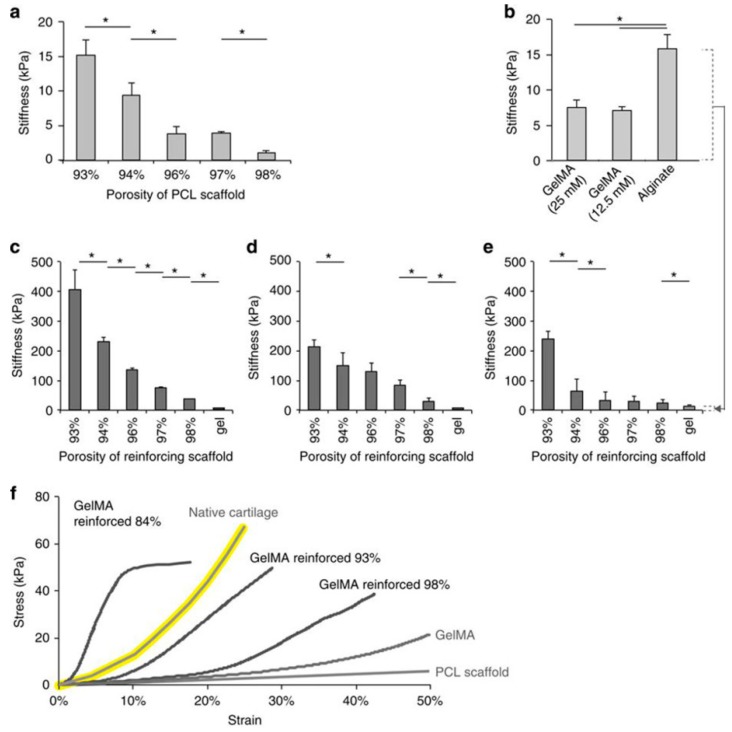
(**a**) Compressive moduli of PCL scaffolds; (**b**) hydrogels alone. GelMA reinforced with PCL scaffolds and crosslinked with either (**c**) 25 mM or (**d**) 12.5 mM APS/TEMED were one order of magnitude stiffer than the scaffolds or gel alone; (**e**) a comparable degree of reinforcement for reinforced alginate gels; (**f**) Stress–strain curves of GelMA, the PCL scaffold and reinforced GelMA, approaching the curve of native cartilage (yellow) [98]. (Reproduced with permission from Visser J et al. Nature Communications; published by Springer Nature, 2015).

However, the application of GelMA hydrogel in bone tissue engineering is limited by its mechanical strength. Many strategies have been devoted to improving the mechanical strength of hydrogel. Adding rigid materials is a way to improve the poor mechanical strength of GelMA hydrogel. Shin H et al. [73] incorporated rigid and brittle gellan gum methacrylate (GGMA) into soft gelatin methacrylamide (GelMA), and prepared double-network (DN) hydrogels with enhanced mechanical strength. Zuo YC et al. [46] attempted to mix methacrylated gelatin (GelMA) with hydroxyapatite (HAP). They successfully prepared GelMA–HAP hybrid hydrogel, and the mechanical strength of GelMA–HAP hybrid hydrogel was stronger than pure GelMA hydrogel, meanwhile, hybrid hydrogel possessed better biocompatibility and swelling ability. They also found that 3D scaffold prepared by GelMA–HAP hybrid hydrogel could promote cell growth and differentiation. These results showed that GelMA–HAP hybrid hydrogel had wide prospects in bone regeneration applications. Recently, Wang Y et al. [53] also prepared GelMA/PEGDA hydrogel with improved mechanical properties. Using microfibre networks is another method for mechanical strength enhancement. Visser J et al. [98] used poly(ɛ-caprolactone) (PCL) fiber scaffolds to increase the mechanical stiffness of GelMA hydrogels (Figure 3). Covalent binding between gel and scaffold can also facilitate enhanced mechanical strength. Boere KWM et al. [74] obtained hydrogels with enhanced mechanical properties by covalently binding the GelMA hydrogel to a thermoplastic polymer network of poly(hydroxymethylglycolide-co-ɛ-caprolactone) (pHMGCL)/PCL and methacrylated pHMGCL (pMHMGCL)/PCL.

**Figure 4:**
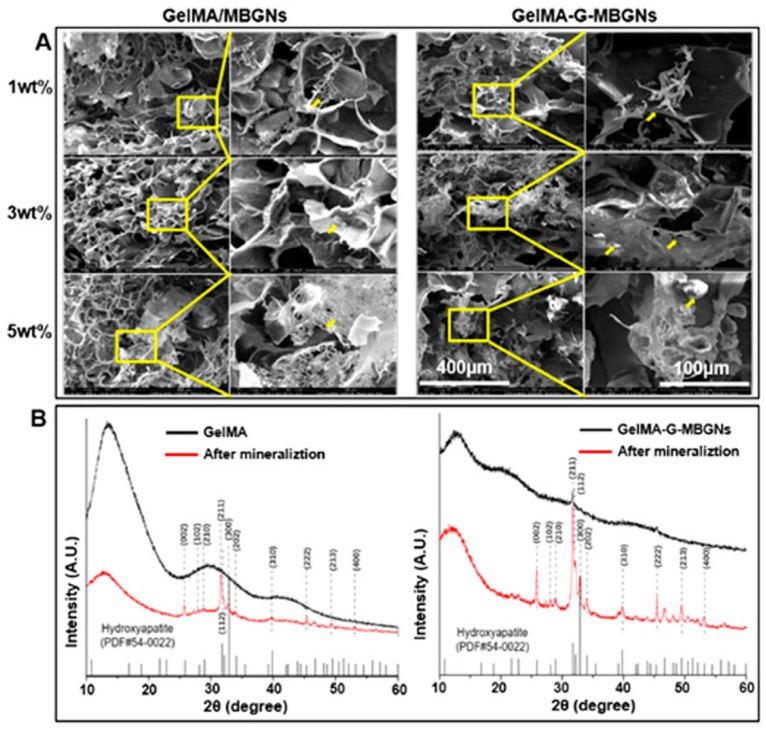
(**A**) SEM images of GelMA/MBGNs and GelMA-G-MBGNs after soak in SBF in low magnification and high magnification. (**B**) XRD images of GelMA and GelMA-G-MBGNs after soak in SBF [101]. (Reproduced with permission from Xin T et al. ACS Applied Materials & Interfaces; published by American Chemical Society, 2017).

Mineralization is one of the most important characteristics of bones. Bone mineralization is a physiological process during which the osteoblasts secrete organic or inorganic components that make up the extracellular matrix. Therefore, mineralization is another important research branch in bone tissue engineering. Studies on GelMA hydrogels-based bone mineralization have been carried out. Zuo Y et al. [99] proposed that the building of osteon-like structures in bionic bone is a great challenge for mimicking the native bone tissue. They successfully developed a circle-and-cross method and a layer-by-layer method to build osteon-like structures. Compared with the circle-and-cross method, materials produced with the layer-by-layer method possessed better bioactivity. Zhou L et al. [100] reported that the mineralization outcome of the GelMA hydrogel could be controlled by the degree of methacrylation, and the influence of mineralization on mechanical properties was also studied. Mesoporous bioactive glasses nanoparticles (MBGNs) and GelMA were combined to enhance the mineralization by Xin T et al. [101]. They found that the thickness of the mineralization layer could be increased by rising the ratio of MBGNs (Figure 4A). The results of XRD showed that the mineralization degree of GelMA hydrogel had been increased by adding MBGNs (Figure 4B). 

Analogously, GelMA hydrogels have also been widely used in cartilage tissue engineering. The regeneration and self-healing abilities of cartilage are limited, and deficiencies are concomitant with the existing methods for the repair of cartilage defects, which causes the rise of cartilage tissue engineering. The mechanic, swelling and lubricating properties of GelMA hydrogels are similar to natural cartilage [102]. The cells that make up the cartilage tissue are chondrocytes only, therefore, the influence of GelMA hydrogel on chondrocytes has caused concern among researchers. For example, Li X et al. [103] pointed out that the GelMA hydrogel stiffness could affect the chondrocyte phenotype. In this study, the stiffness of GelMA hydrogel was changed by adjusting the degree of substitution of GelMA hydrogels. They found that GelMA hydrogels with high stiffness allowed for maintaining the phenotype of chondrocytes. Subsequently, Li X et al. [104] studied the influence of microporous structures of GelMA hydrogel on chondrogenesis. A higher proliferation rate was observed with microporous GelMA hydrogels, and GelMA hydrogels without a microporous structure showed clear advantages on cartilaginous phenotype of chondrocytes. Mouser VHM et al. [80] studied the influence of spatial chondrocyte distribution on the repair process of cartilage defect. Four repair conditions based on GelMA hydrogel were defined and compared, the well-integrated cartilage-like tissue that completely filled the defect was observed, and the results showed that the spatial chondrocyte distribution played an important role in the repair process.

Mechanical strength is critical for scaffold materials which are used to repair cartilage defect. The mechanical strength of GelMA hydrogels is tunable to hold the encapsulated cells, but their mechanical strength is still not comparable to natural cartilage. Therefore, composite GelMA scaffolds were developed to overcome this defect. Levett PA et al. [105] improved mechanical properties of GelMA hydrogel and chondrogenesis by adding glycosaminoglycans (GAGs) into GelMA hydrogel. In this study, Methacrylated hyaluronic acid (HA) and chondroitin sulfate (CS) (HA–MA and CS–MA) were used to achieve the incorporation of GAGs into GelMA hydrogels. The results indicated that GelMA alone could not enable complete chondrogenesis in vitro. However, an instructive environment for the deposition of cartilage-like matrix was provided by GelMA hydrogel added with HA–MA and CS–MA. Bartnikowski M et al. [59] studied GelMA–alginate (ALG)/HAP scaffold and GelMA/HAMA-ALG/HAP scaffold which could improve the mechanical strength. Human articular chondrocytes were encapsulated in hydrogels by 3D printing. They observed that cell activity was not affected by the two types of hydrogel scaffolds. And the use of HAMA could improve chondrogenesis, while HAP did not promote the formation of a zone of calcified cartilage (ZCC).

**Figure 5:**
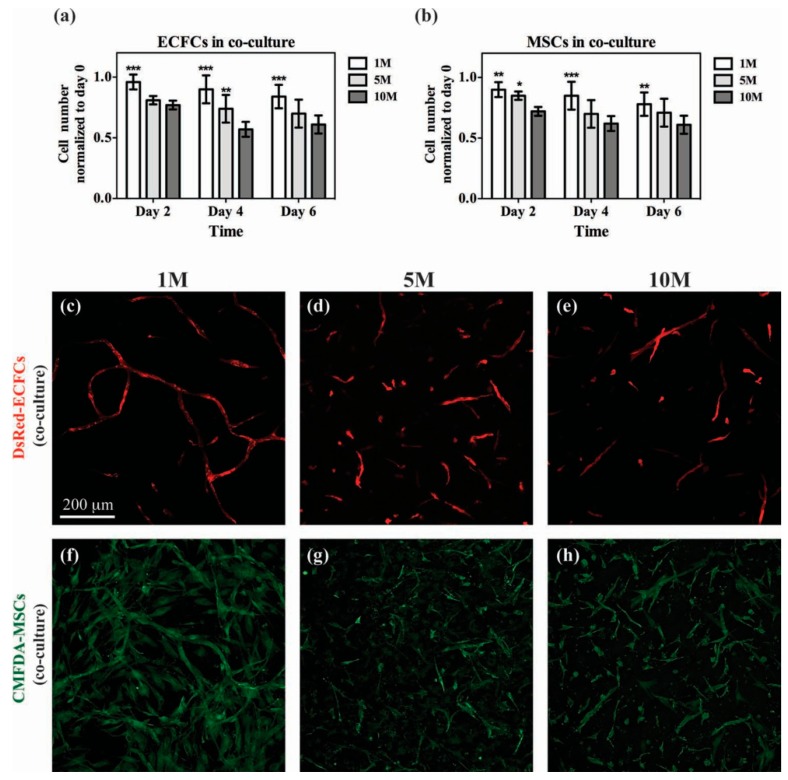
Co-culture of vascular cells in GelMA. Constructs containing both DsRed-ECFCs and CMFDA-labeled MSCs were cultured for 2, 4 and 6 d using GelMA hydrogels with different methacrylation degrees. (**a**,**b**) Numbers of DsRed-ECFCs (**a**) and CMFDA-MSCs (**b**) were separately counted at each time point under a fluorescence microscope. (**c**–**h**) Representative confocal microscopy images of DsRed-ECFCs (**c**–**e**) and CMFDA-MSCs (**f**–**h**) after day 6 in co-culture for each methacrylation degree. [28]. (Reproduced with permission from Chen Y-C et al. Advanced Functional Materials; published by John Wiley and Sons, 2012).

Large bone or cartilage defects repair is necessary in clinical therapy. However, it is difficult to repair bones or cartilage defects with large area mainly because of the insufficient supply of blood, oxygen, and nutrients [103,106]. In order to address this issue, vascular networks were introduced in tissue engineering. Scaffold materials used to form the vascular networks should have suitable network structure and pores. GelMA hydrogels have been used to fabricate vascular networks due to their biocompatibility and structure. Chen Y-C et al. [28] demonstrated the preparation of functional vascular networks using photocrosslinked GelMA hydrogels. They generated capillary-like networks by loading the 3D GelMA hydrogel with human blood-derived endothelial colony-forming cells (ECFCs) and bone marrow-derived mesenchymal stem cells (MSCs) (Figure 5). Subsequently, Lin R-Z et al. [82] injected the solution of GelMA prepolymer (added with human blood-derived ECFCs and bone marrow-derived MSCs) into the subcutaneous space of the mouse, then crosslinked by transdermal exposure to UV light. Vascular networks had then formed within 7 days.

However, it has been a challenge to manufacture large scale bone tissue structures with functional vasculature. Byambaa B et al. [51] proposed an extrusion-based direct-writing bioprinting strategy to manufacture bone-like tissue structures with functional vasculature. The bioprinting of GelMA hydrogels embedded with hMSC and vascular endothelial growth factor (VEGF) enabled the formation of the perfusable blood vessel, and GelMA-hMSC hydrogels loaded with silicate nanoplatelets were printed to act as outer layers, which induced osteogenesis. The results indicated that this strategy could be used for bone regeneration and bone defects repair. However, there is another challenge regarding how to perfuse nutrients in large scale bone tissue scaffolds. Sawyer SW et al. [106] reported casted cell-laden GelMA hydrogels around pre-fabricated 3D printed structures taking polyvinyl alcohol (PVA) as sacrificial materials. Perfusable channels were built within the center of GelMA hydrogels to achieve nutrients perfusion. The model of cell-laden GelMA hydrogels with channels was achieved by 3D printing. This model system can be used for bone tissue engineering.

## 4. Conclusions

GelMA hydrogels can be gained readily by photopolymerization of GelMA prepolymer. Also, the hydrogel properties can be engineered during the preparation processes. Notably, GelMA hydrogels are similar to ECM, and this feature is the basis of further applications in biomedical field. The properties of GelMA hydrogel can be evaluated via various characterization methods (such as H NMR, FTIR, SEM and so on). A wide range of applications can be found with GelMA hydrogels in the field of tissue engineering, including the regeneration or repair of bone, cartilage, neuro, skin, skeletal muscle, cardiac tissues, liver and so on. We mainly reviewed the state-of-the-art research progress of the load-bearing tissue (i.e. bone and cartilage). The research of GelMA hydrogel scaffold with suitable mechanical properties and vascularization for bone and cartilage tissue engineering holds great significance and challenge. In general, three methods were adopted for the enhancement of mechanical properties, including the addition of rigid materials, covalent binding, and fabricating microfibre networks. Vascularization of GelMA hydrogel scaffold is beneficial for the repair of large area bone or cartilage defects. However, little research has focused on microvasculature and larger vascular conduits in 3D bone tissue based on GelMA hydrogels. Despite the applications of GelMA hydrogels appearing to be a long way off from clinical practice, this kind of hydrogel is expected to be one of the most promising candidates for tissue repairing components. Innovative works should be devoted to developing novel preparation methods taking visible light as polymerization energy, and to achieving hydrogels with an increased matching degree between the hydrogels and the tissue defects.

## Figures and Tables

**Table 1:** A summary of researches on GelMA hydrogels in load-bearing tissue and its related tissue.

Tissue	Polymer	Cells	Aims & Achievements	Reference
Bone	GelMA−HA	HUVECs, MG63s	enhanced mechanical rigidity and cell functional expression, modularly engineering of biomimetic osteon.	[46]
	GelMA-nSi	hMSCs	bone regeneration without any osteoinductive factors.	[47]
	GelMA-nHAp	preosteoblasts	increased mechanical stiffness and physiological stability, regeneration of the damaged bone tissue.	[48]
	Alginate-GelMA	MG63, HUVECs	simplified osteon mimicking.	[49]
	Gel/(nHAp-BMP-2)	BMSCs	increased organic/inorganic compatibility, sustainable bioactivity.	[50]
	GelMA	HUVECs, hMSCs	microstructured bone-like tissue constructs containing a perfusable vascular lumen.	[51]
	AlgMA-GelMA	MG63s, HUVECs	osteon-like structure by sequential assembly.	[52]
	GelMA/PEGDA	MC3T3-E1	bone regeneration and enhanced performance.	[53]
	BG/GelMA	mBMSCs	improved bioactivity and stability of composites hydrogels.	[54]
	Bio-GelMA	ADSCs	cell carriers for ADSCs, greater osteogenic differentiation of ADSCs.	[55]
	GelMA	MSCs	vascularisation during endochondral bone repair.	[56]
	GelMA	BMSCs	injectable stem cell-Laden GelMA microspheres, rapid osteogenic Tissue Constructs.	[57]
Cartilage	GelMA	meniscus fibrochondrocytes	combination of cell therapy, GelMA hydrogels, and PSL, production of graft tissue and emulation of meniscus collagen bundles.	[42]
	GGMA/GelMA DNs	NIH-3T3	cartilage mimicking.	[73]
	pMHMGCL/PCL- GelMA	chondrocytes	reinforced GelMA constructs.	[74]
	PEG-GelMA-HA	DPSCs	enhanced chondrogenesis in DPSCs.	[75]
	PEG-GelMA	hMSCs	enhanced mechanical strength.	[76]
	G-MeHA and G-MeCS	chondrocytes	phenotypic stability and integrated cartilage tissues.	[77]
	GelMA/PAM	chondrocytes	improved mechanical property and sustained release of growth factors.	[78]
	GelMA-HepSH	chondrocytes	promoted cell viability and chondrocyte phenotype.	[79]
	GelMA/gellan	chondrocytes	spatial position of chondrocytes in hydrogels and thus in defects.	[80]
	PEGDA/GelMA	MSCs	integration of living cells, biomaterials, and biological cues.	[81]
endochondral bone	GelMA/CDM	MSCs	endochondral bone formation and relevant-size bone grafts.	[58]
	GelMA/HAMA-ALG/HAP	chondrocytes	improved mechanical strength and chondrogenesis.	[59]
vascular networks	GelMA	ECFCs and MSCs	generation of functional and 3D vascular networks.	[28]
	GelMA	ECFCs and MSCs	photopolymerization in vitro and surgical transplant in vivo.	[82]
	PEG–GelMA	HUVECs	reproduction of the extracellular environment.	[83]
	GelMA	MC3T3	fabrication of microchannel networks, promoted cellular viabilityand differentiation, and the formation of endothelial monolayers.	[84]
	GelMA	HUVECs	replicate of geometry and function of vascular networks and blood vessels.	[85]
	GelMA, sodium alginate, and PEGTA	ECs, MSCs	improved transport of oxygen, nutrients, and waste products.	[86]
	GelMA-alginate	ECs	generation of a scaffold with multilayer interlacing hydrogel microfibers	[87]
	GelMA	SCAP, HUVECs	photopolymerization under an LED-light source, and promoted vasculature network formation.	[88]
	GelMA/PCL	ECs	improved endothelium remodeling.	[89]
skeletal muscle	GelMA	C2C12	3D arrays of engineered muscle tissue.	[90]
	GelMA-CNT	C2C12	anisotropic electrical conductivity and superior mechanical properties.	[91]
	GelMA	C2C12	structures and the degree of alignment of myotubes.	[92]
	GelMA	C2C12	enhanced myotube maturation and functionality.	[93]
